# COVID-19 vaccine hesitancy in patients with mental illness: strategies to overcome barriers—a review

**DOI:** 10.1186/s42506-022-00102-8

**Published:** 2022-01-21

**Authors:** Ebrahim Payberah, Daniel Payberah, Ashish Sarangi, Jayasudha Gude

**Affiliations:** 1 Texas Tech University Health Sciences Center School of Medicine, Lubbock, USA; 2 North Shore LIJ-Zucker Hillside Hospital, Glen Oaks, USA

**Keywords:** Vaccine hesitancy, Acceptance, Vaccine refusal, Mental illness, COVID-19, Pandemic

## Abstract

**Background:**

People with mental health problems are at particular risk both for infection with COVID-19 and for more severe course of illness. Understanding COVID-19 vaccine hesitancy is crucial in promoting vaccine acceptance among people with mental health diagnoses. This review aims to identify the prevalence and discuss factors associated with COVID-19 vaccine hesitancy among the mentally ill population.

**Main body:**

We conducted a detailed literature search and included 15 articles for discussion in this review. Several studies showed varying trends of vaccine hesitancy rates among different countries. Major factors involved in vaccine hesitancy in general include mistrust, misinformation, believing in conspiracy theories, and negative attitudes towards vaccines. It was surprising that none of the studies were focused on vaccine acceptance rates and factors associated with vaccine hesitancy among the mentally ill population. However, studies do show that COVID-19 is associated with worse healthcare outcomes for psychiatric patients, and vaccine hesitancy correlated with a lower likelihood of receiving mental health treatment and vaccinations. Psychiatrists need to address issues among patients who are particularly vulnerable to the fear of vaccines which include anxiety, panic attacks, certain phobias including trypanophobia and agoraphobia, obsessive-compulsive disorder, and certain types of traumas. Psychiatrists need to communicate effectively, show respect, empathy, and deliver accurate and honest information about the vaccines. Motivational interviewing, getting people with mental health illness to organize vaccine campaigns, and involving families with mental health problems may promote vaccine acceptance among this group.

**Conclusion:**

Existing literature on the rates of vaccine hesitancy among people with mental health illness is limited. The mental health illness may increase the risk of hesitancy especially in patients having certain emotional disorders such as anxiety and phobia. More studies addressing vaccine hesitancy rates and factors associated with the mentally ill population need to be done in the future.

## Background

Severe acute respiratory syndrome coronavirus 2 (SARS-CoV-2, otherwise known as Coronavirus Disease 2019 or COVID-19) is largely considered to have spread in China in 2019, ultimately becoming a global pandemic by March 2020 [1–4]. Although the exact pathophysiology of COVID-19 is still being investigated, research shows that symptoms arise through coagulopathy, endothelial dysfunction, and inflammatory pathways [1]. While the most heavily reported pathological findings to include alveolar damage with cell membrane hyalinization and immune cell invasion, ultimately leading to the respiratory symptoms of acute respiratory distress syndrome, pneumonia, rhinorrhea, and sore throat—other systemic findings include but are not limited to fever, headache, myalgia, rash, kidney injury, diarrhea, and cardiac abnormalities [1, 5, 6].

Although the COVID-19 pandemic has had large-reaching effects on many populations, one overlooked group is the mentally ill population, who have been disproportionally affected by the pandemic [6–11]. Those suffering from mental illness tend to have more comorbidities—such as obesity, cardiovascular disease, diabetes, and respiratory disease—that increase their risk of contracting COVID-19, and the quarantine and associated social changes put this population at a particular disadvantage [7, 9, 11, 12]. In fact, mental illness is shown to confer worse COVID-19 outcomes when corrected for comorbidities [9]. Research indicates that patients with severe mental illness have 2–3 times greater mortality due to COVID-19 than the general public, with special emphasis on schizophrenia spectrum disorders and substance abuse disorders [7–9, 13, 14]. One explanation is that COVID-19 is correlated with increased isolation and loneliness, depression, stress, and insomnia, which can weaken the immune system [9, 12, 15, 16]. It is also likely that increased financial difficulties and access to mental health resources worsen the health outcomes for mental patients [7, 9, 16]. Moreover, some studies claim that COVID-19 has increased both the incidence of psychosis as well as relapses of psychotic symptoms [2, 13]. The occurrence of the pandemic has further worsened the burden of mental health, where there is already an increase in the mental illness in children and adolescent populations [16].

With the devastating impact that COVID-19 places on multiple communities, treatments are imperative. Currently, many acute treatment plans show very limited efficacy, and officials agree that preventative medicine—such as vaccinations—is the most effective and highest priority long-term measure, especially for lower-income countries [1, 17, 18]. Despite this, a significant challenge to public safety is vaccine hesitancy, or the reluctance or refusal to vaccinate oneself or one’s children despite the availability of vaccines and studies validating their safety and efficacy [10, 19, 20]. This study aims to understand vaccine hesitancy for modern COVID-19 vaccines among mental illness populations.

## Main text

Articles addressing COVID-19 vaccine hesitancy among mental illness patient populations were queried in PubMed and SCOPUS using terms including “vaccine”, “hesitancy”, “COVID-19”, “mental disorders”, and “psychological”. Researchers selected 13 articles from PubMed, 1 article from SCOPUS, and 1 article from other sources after duplicates were removed. Letters to the editor, commentaries and book chapters were excluded from our search.

Overall, studies show that vaccine acceptance rates vary among different countries and time of conducting the study in relation to the status of roll out of vaccines [4, 21]. A systematic review shows that vaccine acceptance rates in the USA are among the lowest at 56.9%, with vaccine hesitance or resistance reaching up to 33%. This review reports also that the lowest vaccine hesitancy rates (defined as less than 60% acceptance) is found in countries such as Kuwait, Jordon, Italy, Russia, Poland, the USA, and France [21]. Another study focusing on UK and Irish populations indicated a rate of vaccine hesitance and resistance of about 30% [4].

Observing demographic factors more closely, studies reported multiple variables affecting vaccine hesitancy. Studies indicate that females show higher vaccine hesitancy [4, 21, 22]. American studies have shown that up to 36% of surveyed African American and Hispanic minorities have vaccine hesitancy, higher than the general public [20, 22, 23]. Living in rural areas, as well as lower levels of economic status, education, and employment correlated with higher vaccine hesitancy [4, 22]. Moreover, vaccine hesitancy extends into the healthcare workforce, with one Israeli study showing little difference in vaccine hesitancy between physicians and the general population [24]. Likewise, a study of vaccine hesitancy among healthcare workers in several middle eastern countries found that 40.1% of the studied population was unsure of getting the vaccine, with another 19.2% declining entirely [25]. Another systematic review found that one-fourth of included studies reported negative attitudes towards the COVID-19 vaccine among healthcare workers [26]. Another study showed almost a quarter of surveyed medical students from an allopathic medical school in Michigan were not willing to immediately take a COVID-19 vaccine upon FDA approval [27]. Nonetheless, one Canadian multicenter study found a high vaccine acceptance rate among healthcare workers to be nearly 80% [28]. Notably, there exists a correlation between willingness to take the COVID-19 vaccine and previous vaccination for the influenza vaccine in the same year [29].

While sources of vaccine hesitancy are still being investigated, multiple studies indicate that misinformation and conspiracy beliefs significantly influence vaccine hesitancy in the general public [12, 20, 23, 27]. One study found a significant correlation between vaccine-resistant participants and the likelihood of getting news on COVID-19 from social media, as opposed to traditional news outlets, researchers, and government agencies [4]. Likewise, news on COVID-19 seems to disproportionately report rare events related to COVID-19 vaccines, and political polarization in the US is a strong predictor of vaccine hesitancy [22, 23]. Other possible explanations for vaccine hesitancy may lie with the expedited development of recent vaccinations, with a subsequent lack of information generating public caution in vaccine safety, despite following standard protocols for vaccine development [10, 17, 19, 20, 23, 24, 30].

Negative emotions influence vaccine hesitancy, in large part due to the changes to public life, socioeconomic upheaval and uncertainty, and loss of loved ones among other factors [17]. As previously stated, rapid vaccine development, misinformation, and reliance on social media for news on COVID-19 have evoked emotions of concern, fear, and distrust [4, 16, 17, 19, 23, 24]. The pandemic has resulted in an increase in anxiety and depressive symptoms. A systematic review and meta-analysis focusing mostly on Chinese studies reported almost half of the general population seeing worsening psychological impacts of COVID-19, and up to 40% reporting bad sleep quality, which correlates with worse immune health [12]S. In fact, one review shows that short-term and long-term stressors among the general population reduce vaccination response [15]. A study on the Irish and UK populations reported psychological indicators of vaccine hesitancy and resistance to include less trust in scientific and governing authorities, negative attitudes towards migrants, higher social dominance, lower cognitive reflection, lower altruism, lower agreeableness, lower conscientiousness, lower emotional stability, higher impulsivity, higher neuroticism, higher paranoia, higher prevalence of conspiracy beliefs, and a higher reported internal locus of control [4]. Regarding the latter two factors, it’s possible that conspiracy beliefs may be a mechanism of improving a sense of control during concerning and rapidly evolving times [23]. Individual perceived-risk also plays a role in behavior during this pandemic. One survey of the general American population found that the groups who perceived a higher risk of COVID-19 had a higher acceptance rate for the vaccine [31]. Another Finnish survey found that while participants did not perceive a great risk towards their own personal health, they viewed COVID-19 as a severe risk towards the general public, and correspondingly reported a 73.9% vaccine acceptance rate if the vaccine was recommended by authorities [30]. These studies show that with greater perceived risk of the disease, individuals are more likely to engage in recommended preventative measures [21, 24, 30]. Finally, one study reported that needle phobia may affect anywhere from 3.5 to 20% of the population [3].

With a focus on mental illness patients, it is important to note that there is a scarcity of studies regarding vaccine hesitation [9, 13]. Reports show that vaccine hesitancy correlates with a lower likelihood of receiving mental health treatment and vaccinations, exacerbating discrepancies between mental-illness and general populations for the COVID-19 pandemic [4, 9–11]. One pre-COVID-19 American study attempting to improve vaccine acceptance among mental illness patients cited vaccine-education, costs, and access to healthcare as barriers to vaccine acceptance [32]. Notably, this same study found that addressing these barriers among mental-illness patients improved attitudes about the safety and efficacy of immunizations from 82 to 94%, with intention to receive CDC-approved vaccinations rising from 58.4 to 93.8% [32]. Armed with guidance from this study, various stakeholders may join hands to improve vaccine acceptance by establishing readily available access to healthcare education and public funded vaccine drives.

As hypothesized, studies show that COVID-19 is associated with worse healthcare outcomes for mental illness patients [7, 9, 32]. Patients with schizophrenia are at higher risk for COVID-19 transmission and mortality, and antipsychotic medications such as Clozapine may even be implicated in a weaker immune system for these patients [7, 9]. Likewise, the increased incidence of depressive symptoms, isolation, anxiety, and insomnia put individuals with depressive disorders at higher risk, as depression is historically correlated with weaker vaccination against other diseases such as influenza, measles, hepatitis B, herpes zoster [9, 12, 15]. Furthermore, with the increased incidence of smoking, heavy drinking, and other emotional disturbances during the COVID-19 pandemic, people with substance use disorders are also disproportionately affected—especially with structural barriers to receiving proper healthcare in social isolation [14, 15].

## Discussion

Mental health professionals play an important role to increase the uptake of vaccines among people with mental health diagnoses. However, mental health professionals face a lot of challenges when patients with mental health illness express vaccine hesitancy. Among mental health disorders that are particularly vulnerable to the fear of vaccines are anxiety, panic attacks, and certain phobias including trypanophobia and agoraphobia, obsessive-compulsive disorder, and certain trauma. Patients with agoraphobia may not want to drive or use subways or other public transportation to travel to receive a vaccine, fears that may be multiplied by the chance of getting COVID-19 once they leave the house [33]. Patients with obsessive-compulsive disorder might also be hesitant about receiving vaccines with the fear of getting infected/injected with germs. Some patients may be afraid of needles (trypanophobia), according to a meta-analysis done by McLenon et al. the prevalence of trypanophobia was 30% among young adults [34]. This fear might further enhance anxiety about receiving the COVID-19 vaccine. Patients with severe mental illness may also experience barriers to immunization, including a lack of knowledge and awareness, accessibility problems, costs, fears about immunization, and often no recommendations from their primary care providers [32]. In addition, patients experiencing paranoid delusions may not trust vaccines. They may reject science, distrust the way vaccine is produced, and distrust its efficacy, fear about outcomes, and fear about interacting with psychotropic medications. They may also feel the government is injecting devices to track them in the name of the vaccines. People with grandiose delusions may express they are immune to coronavirus and not feel the need for receiving the vaccine. On the other hand, people with depressive disorders may express vaccine hesitancy because of a lack of energy and motivation. Individuals with suicidal ideations may also express minimal motivation and not realize the importance of receiving the vaccine. Different kinds of myths about COVID-19 have been rumored around in social media like COVID-19 vaccine gives you COVID, or COVID-19 vaccine changes the DNA, induces infertility, the vaccine may induce psychosis, or insert a microchip in the body [35, 36]. Mistrust and misinformation may further fuels paranoia and increase anxiety resulting in vaccine hesitancy and avoidance.

Psychiatrists play a very crucial role as they serve as a trusted point of contact between patients with mental illness and the general medical system. Information with patients must be shared in a way that is appropriate for a patient’s language, cultural, and educational background. Psychiatrists and other healthcare professionals treating people with severe mental illness should be aware and knowledgeable of the different types of vaccines that become available, their safety and efficacy for their patients, and the applicable vaccination schemes. It is the responsibility of psychiatrists to reach out to their patients and provide them with the best possible care [37]. The American Psychiatric Association recommends that psychiatrists should actively engage, discuss this topic with their patients, address their concerns and give accurate information. Psychiatrists should also assist primary care providers in determining individual decision-making capacity to provide informed consent for vaccination [38]. In this information age, the internet and social media have led to a lot of misinformation putting out information with little fundamental understanding of the performance and value of vaccines. News coverage may further increase paranoia about vaccines in people with schizophrenia. It becomes important for the psychiatrist to establish rapport and educate the patients regarding the vaccines. People with mental health illnesses may also express fear about the efficacy of vaccines, if it may cause psychosis or result in a relapse of psychosis. It is very crucial to provide accurate information to them. Although little is known about the interaction of the COVID 19 vaccine with psychotropic drugs, it is important to provide up-to-date information. Psychiatrists should try to motivate people with mental health problems to be involved in designing immunization programs, addressing concerns about vaccines, and build trust, confidence, and acceptance of vaccines. Motivational interviewing aims to support decision-making by eliciting and strengthening a person's motivation to change their behavior based on their own arguments for change. Motivational interviewing calls for a respectful and empathetic discussion of vaccination and helps to build a strong relationship [39].

A targeted vaccination program focusing on people with mental health problems can result in increasing vaccination rates among this group. Sometimes people with severe psychosis or paranoia may only trust their psychiatrist so it is important that psychiatrists be trained and ready to administer vaccines in case a need arises. It is also important that mental health professionals evaluate the decision-making capacity, negative thoughts regarding vaccines and discuss the risk vs benefits to promote acceptance of vaccines. Psychiatrists should also include parents and families of people with mental illness to promote vaccine acceptance among people with mental health problems. Psychiatrists need to communicate effectively show respect, empathy, and deliver accurate and honest information about vaccines [38]. One randomized controlled trial showed that telling stories and images highlighting the effects of vaccine-preventable diseases improved attitudes toward vaccination, especially for individuals who had lower confidence in vaccines [40]. It is equally important to address any concern about the pain and side effects that may result because of the vaccine which is perceived to be a barrier against effective vaccination strategies. Psychiatrists should collaborate with primary care providers and organize more public health campaigns to spread more awareness about the benefits of vaccines and increase acceptance rates [40]. As various COVID-19 vaccine programs are currently undergoing it is important to address the above concerns to make sure to drive higher vaccine acceptance.

## Conclusion

It is concerning that there are limited studies focusing on the vaccine hesitancy rates and associated factors affecting the vaccination rates among people with mental health problems. Mental health professionals play an important role in promoting vaccine acceptance. It is crucial to building confidence regarding vaccines among people with mental health problems to attain the goal of fighting this global pandemic. More studies addressing these gaps in knowledge need to be done in the future.

## Data Availability

Data sharing is not applicable to this article as no datasets were generated or analyzed during the current study.

## Abbreviations

SARS-CoV-2: Severe acute respiratory syndrome coronavirus 2
COVID-19: Coronavirus Disease 2019
